# Cost-Utility Analysis of Heberprot-P as an Add-on Therapy to Good Wound Care for Patients in Slovakia with Advanced Diabetic Foot Ulcer

**DOI:** 10.3389/fphar.2017.00946

**Published:** 2017-12-22

**Authors:** Tomas Tesar, Laszlo Szilberhorn, Bertalan Nemeth, Balazs Nagy, Martin Wawruch, Zoltan Kalo

**Affiliations:** ^1^Department of Organisation and Management in Pharmacy, Faculty of Pharmacy in Bratislava, Comenius University, Bratislava, Slovakia; ^2^Syreon Research Institute, Budapest, Hungary; ^3^Department of Health Policy and Health Economics, Eötvös Loránd University (ELTE), Budapest, Hungary; ^4^Faculty of Medicine in Bratislava, Institute of Pharmacology and Clinical Pharmacology, Comenius University, Bratislava, Slovakia

**Keywords:** decision making, insurance, health, health policy, Slovakia, reimbursement mechanisms

## Abstract

**Objectives:** To explore whether Heberprot-P (an epidermal growth factor) is a cost-effective option for the treatment of advanced diabetic foot ulcer as an add-on therapy to good wound care (GWC) in Slovakia from the perspective of health care payers.

**Methods:** A Markov model was constructed to compare the costs and effects of Heberprot-P plus GWC to those of GWC alone from the perspective of health care payers. The 52-week clinical trial period was extended to 5- and 10-year time horizons. Transition probabilities were calculated based on a previous clinical trial of Heberprot, utility values were derived from the scientific literature, and cost vectors were collected from the General Health Insurance Fund database in Slovakia. A one-way deterministic sensitivity analysis was employed to explore the influence of uncertainty for each input parameter on the incremental cost-effectiveness ratio (ICER).

**Results:** Based on the ICER threshold of €30,030 per quality-adjusted life year (QALY) recommended by the Slovak Ministry of Health, Heberprot-P therapy plus GWC is not a cost-effective alternative to GWC alone over a 10-year time horizon. The ICER increases if a longer time horizon is applied, as the incremental costs are similar, but the aggregated utility gain from avoided amputation is lower. Based on the sensitivity analysis, the utility multiplier for the health state “no ulcer after small amputation” had the most impact on the ICER; however, the model was robust to changes in all input parameters.

**Conclusions:** Heberprot-P, as an add-on therapy to GWC in the treatment of advanced diabetic foot ulcer, is not a cost-effective alternative to GWC alone. However, if the unit cost of Heberprot-P were to be reduced to <€273, its ICER would be <€30,030.

## Introduction

Core principles of the Slovak health care system include obligatory public insurance, general coverage, and an essential benefits package. Furthermore, the Slovak insurance model promotes competitiveness based on selective contracts with health care providers and flexibility in health services pricing (Smatana et al., 2016).

Davis et al. (2006) highlighted that diabetes mellitus is a non-communicable endocrine disease that leads to serious health complications, such as foot ulcers, and it has a globally increasing incidence. The incidence of lower-extremity amputations ranges from 2.1 to 13.7 per 1000 cases of diabetes (Bartus and Margolis, 2004).

Heberprot-P is a recombinant human epidermal growth factor. It is intended as an add-on therapy to conventional treatment for diabetic patients with neuropathic and ischemic ulcers, at stages 3 and 4 of the Wagner Ulcer Grade Classification System, with a wound area >1 cm^2^. Heberprot-P stimulates the formation of useful granulation tissue, which allows healing by secondary intention or following a skin autograft, as demonstrated in several clinical studies (Fernández-Montequín et al., 2009; Berlanga et al., 2013).

The Slovak Ministry of Health has stated that, despite Heberprot-P not having received marketing authorization from the European Medicines Agency or the Slovak marketing authorization authority (the Slovak Institute for Drug Control), it can be administered to patients in Slovakia. This is an exception to the Slovak legislative Act No. 362/2011, which declares that only medicinal products with marketing authorization can be used for treatment.

Cuba has offered to pay its historic debt to the Slovak Republic using the medicinal products that it can spare, including Heberprot-P. The debt is largely a legacy of business ties between Cuba and Czechoslovakia, which split into the Slovak Republic and the Czech Republic in 1993, 4 years after the end of four decades of communist rule.

In the Slovak Republic, drug reimbursement requires stringent criteria to be met, which involves the evaluation of clinical evidence along with cost evidence, full transparency, and the possibility of a formal appeal (Barnieh et al., 2014). Act No. 363/2011 Coll. (Ministry of Health, 2011a) defines two thresholds (“λ_1_” and “λ_2_”) used by the Slovak Ministry of Health in the reimbursement decision-making process. The lower threshold (λ_1_) is 24 times the average monthly salary, and the upper threshold (λ_2_) is 35 times the average monthly salary (Ministry of Health, 2011a). When the incremental cost per quality-adjusted life year (QALY) is lower than or equal to λ_1_, the drug is fully or partially reimbursed. For cases in which the incremental costs per QALY are higher than λ_1_, but do not exceed λ_2_, the drug is reimbursed with conditions (Ministry of Health, 2011a).

Two options have been considered for the public financing of Heberprot-P for Slovak patients. The first option is to reimburse this medicinal product using health insurance funds. Slovakia has a pluralistic system of health insurance companies, with three health insurance companies operating: the state-owned General Health Insurance Company (“Všeobecná zdravotná poist'ovňa”), and the private Trust (“Dôvera”) and Union, which covered 63.31%, 27.92%, and 8.77% of Slovak population, respectively, in 2015 (Health Care Surveillance Authority, 2016). The second option is to cover Heberprot-P based on Cuba's debt repayment to Slovakia.

The Pharmaceutical Faculty of Comenius University in Bratislava, Slovakia, and the Syreon Research Institute in Budapest, Hungary, were asked by the Slovak Ministry of Health to perform a pharmacoeconomic study concerning Heberprot-P within the settings of the Slovak health care system. The research question concerned whether Heberprot-P is a cost-effective option for treatment of advanced diabetic foot ulcer (DFU) as an add-on therapy to good wound care (GWC) in Slovakia.

## Materials and methods

A Markov model was constructed to compare the costs and effects of Heberprot-P plus GWC to those of GWC alone over a 52-week period based on clinical trial data (Fernández-Montequín et al., 2009). Previous Markov models applied one-year time horizon in the economic evaluation of new technologies in diabetic foot ulcer (Redekop et al., 2003; Gilligan et al., 2015; Guest et al., 2017). According to the local methodological guidelines time horizon of economic evaluations should be long enough to take into account all important clinical outcomes and costs (Ministry of Health, 2011b). As quality of life benefit of avoided amputation stays constant over time, time horizon of the model was extended to capture these benefits over 10 years. In addition, a scenario analysis involving a shorter (i.e., five-year) time horizon was also carried out. A one-week cycle length was chosen, following the structure of the clinical trial data (Fernández-Montequín et al., 2009). According to the Slovak methodological guidelines for economic evaluations, a health care perspective (i.e., third-party payers' perspective) was applied (Ministry of Health, 2011b). A 5% discount rate was applied to both the health gains and costs (Ministry of Health, 2011b).

**Figure 1:**
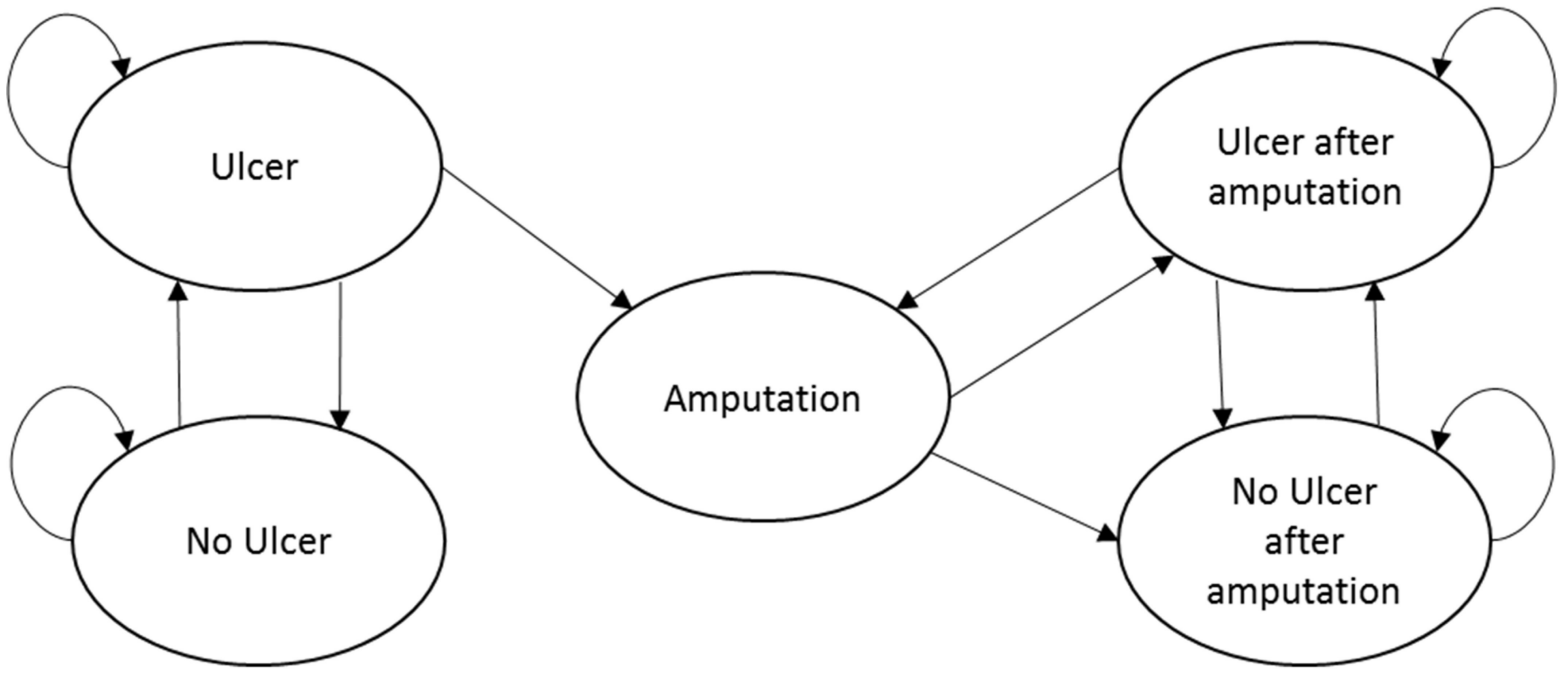
Transition routes of the patients in the Markov model.

The Markov model included five mutually exclusive health states: (1) no ulcer; (2) ulcer (the starting health state for all patients); (3) amputation; (4) no ulcer after amputation; and (5) ulcer after amputation (see Figure 1). We assumed that Heberprot-P has no direct or indirect influence on mortality, and the age- and disease-related mortality of the patients remain constant during the 10-year modeling time horizon. Consequently, we simplified the model structure by excluding death from the health states.

Deep ulcer-related infections (such as osteomyelitis) or gangrene are significant complications of DFU. However, the clinical trial report had no detailed information about these complications; therefore, we did not separate these complications into Markov health states. We assumed that 10% of active ulcers are infected, based on a previous modeling study (Redekop et al., 2003).

### Transition probabilities

In the clinical trial, efficacy results were reported at two, eight, and 52 weeks. Unfortunately, at 2 and 8 weeks, the key efficacy outcome was >50 and >75% granulation, respectively. Therefore, no information was available about how many patients had a fully-healed ulcer (i.e., “no ulcer”) at these time points. Consequently, we calibrated weekly transition probabilities to reflect the 52-week efficacy parameters (Fernández-Montequín et al., 2009), including: (1) ulcer closure; (2) amputation; and (3) ulcer recurrence.

We assumed that at 8 weeks, Heberprot-P therapy had no further benefit beyond the 52-week observation period, so between years one and 10, the transition probabilities of the GWC arm were applied to both arms. We also assumed that ulcer recurrence and healing rates were equal in both arms after amputation. Recurrence in the GWC arm was estimated by dividing the two recurrent cases by the total population in both arms (108 patients).

### Costs

Cost data for the health states used in the model were provided by the Slovak Ministry of Health, which retrieved them from the database of the largest (63.31% of the population) Slovak health insurance company, General Health Insurance Company. The Heberprot-P treatment cost was calculated based on the unit cost per injection multiplied by the number of units needed per week and the length of therapy (in weeks).

### Utilities

The utilities associated with the health states (see Table 1) were based on a published research paper that used the time trade-off methodology (Redekop et al., 2004). We assumed, based on the database of the General Health Insurance Fund, that 50% of minor amputations are toe amputations and the remaining 50% are foot amputations, while all major amputations are leg amputations. Annually, there are 500–600 major amputations (legs) and 3,000–5,000 small amputations (part of feet or toes) for diabetic patients in Slovakia.

**Table 1:** Utilities associated with health states.

**Health state**	**Utility**	**Source**
Diabetic patient with no ulcer (reference value)	0.840	Redekop et al. (2004)
Ulcer (multiplier)	0.890	Redekop et al. (2004)
Infected ulcer (multiplier)	0.820	Redekop et al. (2004)
After small amputation, no ulcer (multiplier)	0.830	Mean of patients with toe or foot amputation, based on Redekop et al. (2004)
After leg amputation, no ulcer (multiplier)	0.730	Redekop et al. (2004)
Acute 30-day post-amputation period (multiplier)	0.500	Assumption
Infected ulcer after small amputation (multiplier)	0.715	Mean of patients with toe or foot amputation and infected ulcer, based on Redekop et al. (2004)
Infected ulcer after leg amputation (multiplier)	0.620	One leg amputated and active infected ulcer, based on Redekop et al. (2004)
Proportion of infected ulcers out of all ulcers	10.00%	Assumption based on Redekop et al. (2003)
Proportion of leg amputations out of all amputations	12.08%	Slovak Ministry of Health data

A one-way deterministic sensitivity analysis was employed to explore the influence of uncertainty for each input parameter on the incremental cost-effectiveness ratio (ICER). All the parameters were changed by ±10%.

Probabilistic sensitivity analysis is not part of the routine economic evaluation in Slovakia (Ministry of Health, 2011b).

## Results

Over a 10-year time horizon, Heberprot-P generated a QALY gain of 0.1709 at an incremental cost of €15,440 (see Table 2).

**Table 2:** Incremental cost-effectiveness ratio (ICER) based on a 10-year time horizon.

	**Heberprot-P plus GWC**	**GWC**	**Difference**
QALY	6.5610	6.3901	0.1709
Cost (€)	130,675	115,235	15,440
ICER (€ per QALY)	90,344		

*QALY, quality-adjusted life year; ICER, incremental cost-effectiveness ratio; GWC, good wound care*.

The ICER worsened when a shorter time horizon was applied, as the incremental costs remained similar, but the aggregated utility gain from avoided amputation was lower (see Table 3).

**Table 3:** Incremental cost-effectiveness ratio (ICER) based on a five-year time horizon.

	**Heberprot-P plus GWC**	**GWC**	**Difference**
QALY	3.6784	3.5814	0.0970
Cost (€)	81,167	65,727	15,440
ICER (€ per QALY)	159,227		

**Figure 2:**
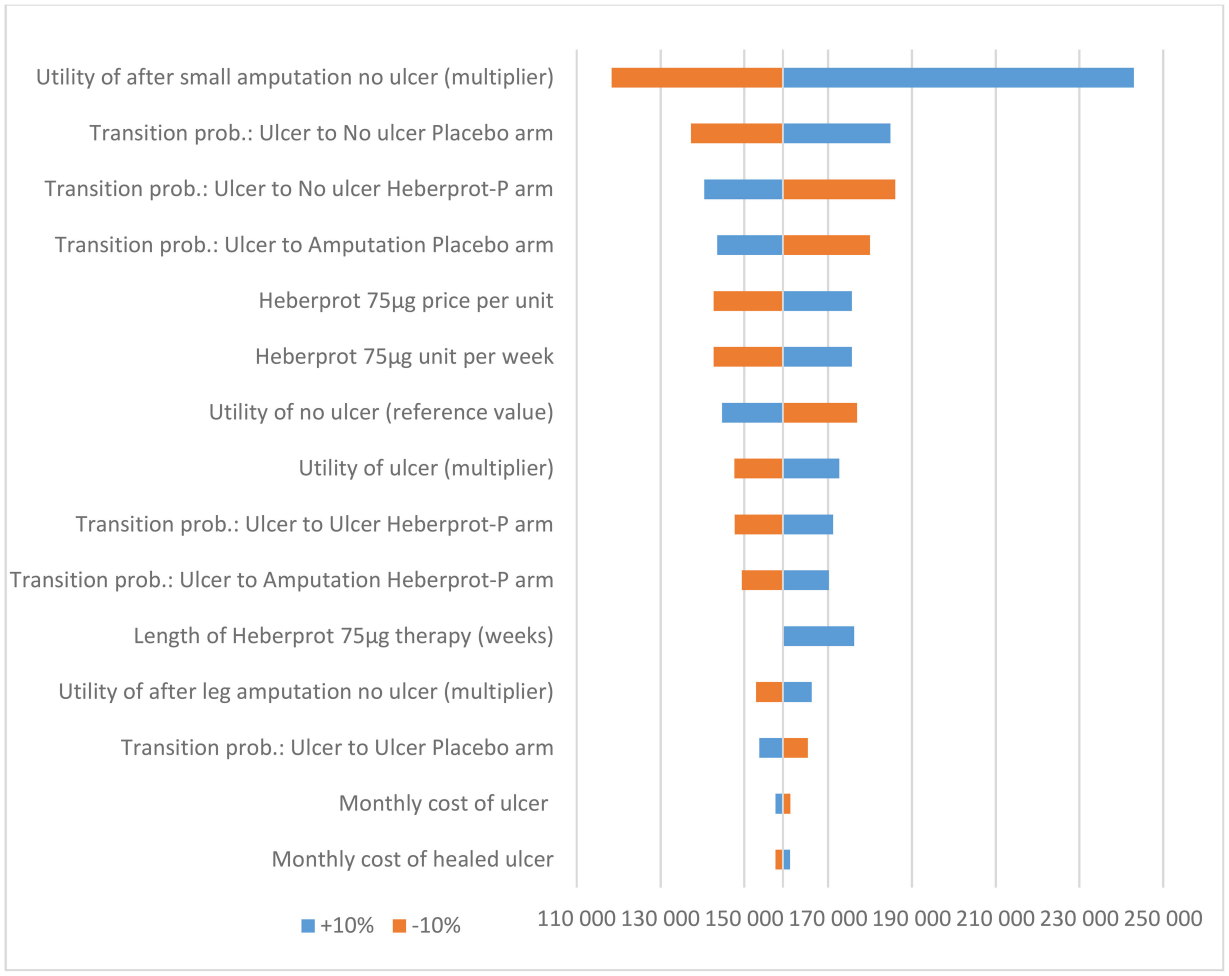
Results of the deterministic sensitivity analysis (5-year time horizon) in €.

**Figure 3:**
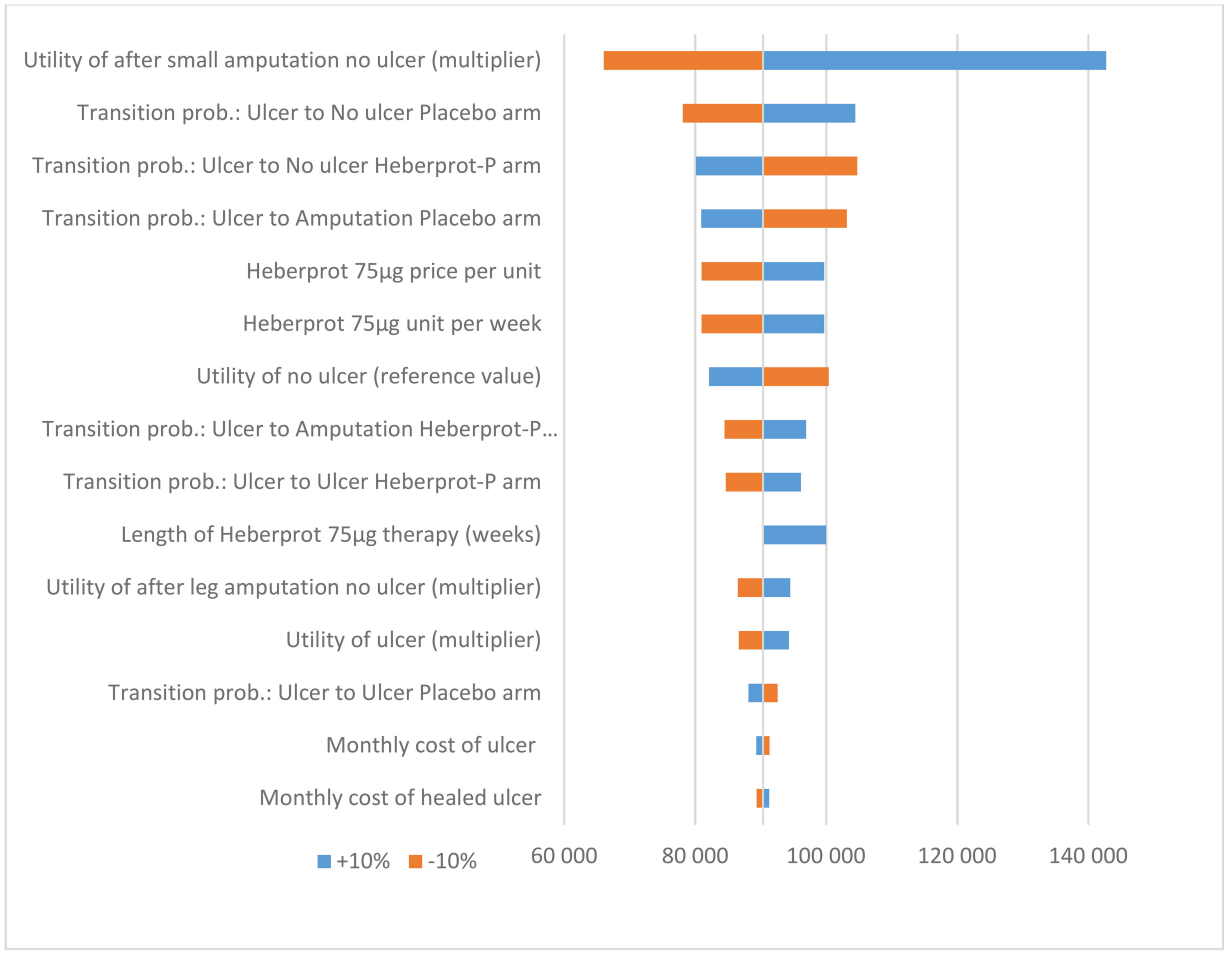
Results of the deterministic sensitivity analysis (10-year time horizon) in €.

Based on the sensitivity analysis, the utility multiplier for the health state “no ulcer after small amputation” had the most impact on the ICER. However, the model was robust to changes in all input parameters (i.e., the conclusion did not change when the input parameters were each changed by ±10%) (see Figures 2 and 3).

The results of this pharmacoeconomic study of Heberprot-P were validated by the Slovak Ministry of Finance.

## Discussion

Kaló et al. (2008) identified a number of potentially cost-ineffective medicines that were being reimbursed prior to 2008 in the Slovak Republic. Since 2011, economic criteria have been applied to pharmaceutical pricing and reimbursement decisions, with explicit thresholds defined by Slovak legislation. Consequently, funds allocated to new medicines reflect opportunity costs in the Slovak health care system (Tesar et al., 2017).

Act No. 363/2011, Coll. (Ministry of Health, 2011a) mandates that pharmacoeconomic reports be produced for medicinal product reimbursement decision-making processes. Moreover, the Ministry of Health Decree No. 422/2011 (Ministry of Health, 2011b) stipulates that “pharmaceutical reimbursement decisions must be substantiated with pharmacoeconomic analyses.”

In 2016, drugs with an ICER <€20.592 per QALY (lower threshold, λ_1_) were eligible for reimbursement from the public health insurance funds, while drugs with an ICER of €20.592–30.030 per QALY (upper threshold, λ_2_) could be conditionally reimbursed from these funds. Given the available patient access schemes in Slovakia, Van Wilder et al. (2015) argued that the ICER thresholds (i.e., the thresholds for incremental costs per QALY) provide a cost-effectiveness assessment tool for drugs rather than a reimbursement-exclusion regulation.

Foot ulceration is a serious complication of diabetes mellitus that is associated with an increased risk of amputation. Based on the upper ICER threshold (λ_2_, €30,030) set by the Slovak Ministry of Health, Heberprot-P therapy plus GWC is not a cost-effective alternative to GWC alone based on a 10-year time horizon. If the unit cost of Heberprot-P were to be reduced to <€273, its ICER would be <€30,030. Based on this study, it has been decided that Heberprot-P will not be reimbursed from the Slovak health insurance funds. Instead, Heberprot-P can be administered to patients in Slovakia, and the medicine will be provided by Cuba, as part of its debt repayment to Slovakia.

Cost-effectiveness of Heberprot-P has never been presented in any previous publications. However, generalizability of main conclusions is limited. Heberprot-P has not been evaluated by the European Medicines Agency, therefore high level uncertainty related to clinical effectiveness and safety of Heberprot-P need to be emphasized. In addition, heterogeneity of good wound care in different countries or regions represents an important limitation in the transferability of results to other jurisdictions.

## Author contributions

TT, LS, BeN, BaN, MW, and ZK conceived the conception and design of the study. TT and MW contributed in acquisition of data. LS, BeN, BaN, and ZK carried out the data management and pharmacoeconomic modeling. TT prepared the draft of the manuscript. All authors contributed to editing the manuscript and the approved final version was submitted for publication.

### Conflict of interest statement

The authors declare that the research was conducted in the absence of any commercial or financial relationships that could be construed as a potential conflict of interest.
